# A universal primer-independent next-generation sequencing approach for investigations of norovirus outbreaks and novel variants

**DOI:** 10.1038/s41598-017-00926-x

**Published:** 2017-04-11

**Authors:** Jannik Fonager, Marc Stegger, Lasse Dam Rasmussen, Mille Weismann Poulsen, Jesper Rønn, Paal Skytt Andersen, Thea Kølsen Fischer

**Affiliations:** 1 Virology Surveillance and Research Section, Department of Microbiological diagnostics and Virology, Statens Serum Institut, Copenhagen, Denmark; 2 Department of Microbiology and Infection Control, Statens Serum Institut, Copenhagen, Denmark; 3 Department of Infectious Diseases and Centre for Global health, Clinical Unit, University of Southern Denmark, Odense, Denmark; 4 Department of Veterinary Disease Biology, University of Copenhagen, Copenhagen, Denmark

## Abstract

Norovirus (NoV) is the most common cause of non-bacterial gastroenteritis and is a major agent associated with outbreaks of gastroenteritis. Conventional molecular genotyping analysis of NoV, used for the identification of transmission routes, relies on standard typing methods (STM) by Sanger-sequencing of only a limited part of the NoV genome, which could lead to wrong conclusions. Here, we combined a NoV capture method with next generation sequencing (NGS), which increased the proportion of norovirus reads by ~40 fold compared to NGS without prior capture. Of 15 NoV samples from 6 single-genotype outbreaks, near full-genome coverage (>90%) was obtained from 9 samples. Fourteen polymerase (RdRp) and 15 capsid (cap) genotypes were identified compared to 12 and 13 for the STM, respectively. Analysis of 9 samples from two mixed-genotype outbreaks identified 6 RdRp and 6 cap genotypes (two at >90% NoV genome coverage) compared to 4 and 2 for the STM, respectively. Furthermore, complete or partial sequences from the P2 hypervariable region were obtained from 7 of 8 outbreaks and a new NoV recombinant was identified. This approach could therefore strengthen outbreak investigations and could be applied to other important viruses in stool samples such as hepatitis A and enterovirus.

## Introduction

*Norovirus* (NoV) is a positive-sense single-stranded RNA virus in the *Caliciviridae* family, and at least 40 genotypes divided into seven genogroups have been identified^1–3^. NoV infection is the most widespread cause of non-bacterial gastroenteritis, responsible for up to one fifth of all cases of gastroenteritis globally^4^. Despite some progress^5, 6^, no vaccine or therapeutic intervention is available, and interceptive strategies mainly aim to rapidly identify the source of infection, increase hygiene measures, and isolate infected patients^7, 8^. Conventional molecular analysis of NoV transmission routes relies mainly on standard typing methods (STM) based on Sanger-sequencing of partial RNA-dependent polymerase and capsid genes (RdRp and cap)^9, 10^. More recently, sequencing of larger parts of the capsid gene containing the hypervariable P2 region has increased the discriminatory power to resolve outbreaks with higher accuracy^11, 12^. Although new recombinant or dominant NoV strains has routinely been reported^13, 14^, commonly used gene-specific primers may have limited the ability to rapidly detect emerging strains due to sequence differences in the primer binding regions. In such situations, it is necessary to amplify and sequence these regions using other primers and subsequently to make these sequences publically available to enable other research groups to redesign their primers accordingly. Therefore, the current STM for analysis could lead to incorrect conclusions about possible transmission chains, underestimation of the genetic diversity of NoV, and delay early identification of new emerging strains. Recent methodological approaches including NGS to achieve full norovirus genome coverage have been published. However, these methods mostly rely on genotype specific primers^15–17^ why they, are time consuming and need frequent updating due to the high natural mutation rates of NoV^18^.

Cultivation of pathogens is commonly used to ensure a pure and high concentration for further investigation and has combined with NGS considerably improved the ability to identify transmission chains and resistance genes for cultivated bacterial infections in particular^19, 20^. Although human NoV to some degree can replicate in animal models, no cell culture system exists^21^. Therefore investigation of NoV suspected cases is usually limited to direct analysis of viral RNA in stool samples, in which viral genomes only constitute a minor proportion of the nucleic acids present^22–24^.

Recent studies have addressed several of these problems by using both random sequencing^24, 25^ strategies and different methods for virus enrichment such as: virion isolation and enzymatic removal of host/bacterial nucleic acids^26^, capture-based^27–29^ methods or PCR activated cell-sorting methods^30^. Despite these recent advances, such methods are still time-consuming, laborious and potentially costly and/or rely on approaches that will require extensive periodic updates in primers or probes to reflect the current knowledge on viral diversity.

In this study, we have evaluated an easy-to-use laboratory method that allows for a ~40-fold enrichment of all NoV genotypes in stool samples. Furthermore, we have used bioinformatics approaches to accurately screen for NoV in highly complex samples. Analysis of NoV positive samples from eight foodborne outbreaks yielded sufficient NoV read counts to allow the assembly of several complete or nearly complete genomes for molecular comparisons. Furthermore, this approach allowed for the identification of an additional genotype, missed by STM, as well as discovery of a new recombinant NoV.

## Results

### Using NGS directly on samples

Despite a large sequencing depth allocated to each sample (1.5 to 5.5 million reads), only a relatively small proportion of the obtained reads were of NoV origin (on average: 0.25%; corresponding to ~700 to ~22,000 reads).

### Evaluation of the poly(A)-capture technique

**Figure 1:**
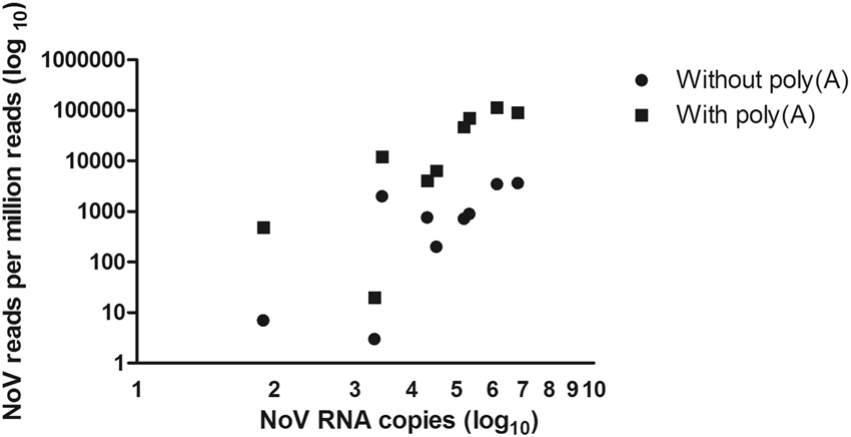
Relationship between the number of NoV RNA copies used as input and the obtained number of NoV reads before and after poly(A) capture NoV input was quantified with real time PCR and GGI and GGI standards and the total number of NoV genome copies used as input was calculated as NoV RNA copies (log_10_) and shown on the X-axis. The number of NoV reads obtained per million reads is shown on the Y axis (log_10_ scale). For each sample, the obtained NoV reads per million reads are shown both with poly(A) capture (filled squares) and without poly(A) capture (filled circles).

To specifically enrich for NoV RNA and reduce the amount of non-polyadenylated bacterial RNA, a poly(A)-capture method was employed after nucleic acid extraction. To evaluate this enrichment strategy, NoV viral load was measured in 6 GGI and 3 GGII quantitated survey samples were (called QS1 to QS9, See Materials and methods and Table 1) along with five non-quantified survey samples (called S1 to S5, See Table 1 and Materials and Methods). All samples were split after RNA extraction with only one part subjected to poly(A)-capture. SMARTer libraries were constructed from both extracted parts and subjected to MiSeq sequencing simultaneously. The efficiency (Table 1) was evaluated by measuring the proportion of reads mapping to full genome sequences from the common human gut bacterial species *Bacterioides uniformis* and *Ruminococcus bromii* L2 + 63^31^ or from a set of 16 sRNA sequences identified in human microbiome studies^32, 33^. Poly(A)-capture increased the proportion of obtained NoV reads over the entire range of NoV input RNA copies (Log_10_ 1,89 to 6,82; see Table 1 and Fig. 1), despite some variation for especially samples with low numbers of input NoV RNA copies. While the proportion of bacterial reads was reduced by 0.28 to 0.41 fold, the number of NoV reads increased by on average 45.1 ± 27.77 -fold. Although the average Ct value decreased by 0.96 ± 0.07-fold after poly(A)-capture, the poly(A)-captured NoV was also eluted in only one fifth of the suspension volume used before poly(A)-capture. The average percentages of reads from the non poly(A)-captured survey samples mapping to three approximately equally- sized parts of the NoV reference genome sequences were: 1^st^ part (genome-position: 1–2499): 32.2% (±17.7%), 2^nd^ part (genome-position: 2500–4997): 53.3% (±12.8%) and 3^rd^ part (genome-position: 4998–7496): 14.5% (±8.9%), while the average percentages of NoV reads from the poly(A)-captured survey samples mapping to these regions were: 1^st^ part: 10.1% (±3.2%), 2^nd^ part: 43.5% (±8%) and 3^rd^ part: 46.4% (±8.9%).

**Table 1:** Summary of quantification, sequencing, mapping and genotyping results of outbreak samples before and after poly(﻿A)-﻿capture.

Sample name	Genotype	Input NoV genome copies (Log10 RNA)	Ct value	Reads in total	Reads after QC and trim	NoV mappings	Norovirus reads/million	B.uniformis reads/million	R. bromii reads/million	16 sRNA reads/million
S1	GII.P21_GII.3	ND	24.54	2,496,612	2,374,862	740	312	255,100	448,900	39,900
S1-poly (A)			23.72	757,000	546,555	7,900	14,454	96,400	173,800	12,400
S2	GI.P3_GI.3	ND	26.3	6,353,762	5,609,338	15,392	2,744	211,200	185,900	90,500
S2-poly (A)			23.96	3,257,730	2,000,579	467,920	233,892	28,600	22,500	7,800
S3	GII.P2_GII.2	ND	24.93	1,572,564	1,555,059	5,969	3,838	530,700	377,500	140,600
S3-poly (A)			21.18	5,260,882	3,233,749	742,233	229,527	202,700	170,500	50,300
S4	GI.Pb_GI.6	ND	26.31	6,034,124	5,659,453	22,362	3,951	192,800	142,600	42,100
S4-poly (A)			23.72	4,037,036	1,992,270	579,005	290,626	97,500	77,900	18,700
S5	GII.P4_New_Orleans_GII.4_Sydney	ND	27.8	2,818,760	2,818,760	15	5	101,800	20,800	5,900
S5-poly (A)			28.04	2,291,272	2,291,247	567	247	13,600	2,100	500
QS1	GI.P2_GI.2	4.52	28	1,156,547	1,100,750	221	201	306,622	438,377	88,697
QS1-poly(A)			27.21	198,236	168,378	1,085	6,444	108,328	200,127	32,635
QS2	GI.P2_GI.2	4.32	28.57	455,397	429,594	328	764	169,900	175,016	35,056
QS2-poly(A)			30.03	113,549	81,662	331	4,053	34,373	42,578	6,392
QS3	GI.P2_GI.2	3.44	31.25	600,614	556,485	1,118	2,009	196,652	341,026	73,608
QS3-poly(A)			28.81	142,905	106,593	1,289	12,093	39,487	51,551	10,770
QS4	GI.P2_GI.2	3.31	31.74	1,492,849	1,381,510	4	3	492,752	298,984	94,092
QS4-poly(A)			35.18	543,183	508,218	10	20	388,874	279,274	70,013
QS5	GI.P2_GI.2	1.89	36	909,479	852,574	6	7	442,575	78,369	29,316
QS5-poly(A)			32.57	171,507	143,530	69	481	179,419	24,838	5,093
QS6	GI.P2_GI.2	6.82	20.97	2,010,472	1,806,439	6,595	3,651	166,246	53,235	25,712
QS6-poly(A)			20.36	385,509	329,523	30,003	91,050	25,889	11,247	4,085
QS7	GII.P16_GII.2	5.34	21.64	3,959,850	3,484,988	3,117	894	398,987	29,031	93,143
QS7-poly(A)			19.03	765,614	560,343	39,653	70,766	44,687	34,522	7,281
QS8	GII.P16_GII.2	6.14	19.2	1,433,289	1,263,790	4,406	3,486	328,972	389,494	113,065
QS8-poly(A)			19.83	655,710	515,093	59,216	114,962	143,494	184,035	80,587
QS9	GII.P16_GII.2	5.20	22.07	2,846,076	2,734,410	1,975	722	458,107	157,780	72,029
QS9-poly(A)			21.28	354,398	284,150	13,302	46,813	38,054	20,419	4,832
Average fold change	NR	NR	0.96	NR	NR	NR	45.13	0.31	0.41	0.28
Standard deviation	NR	NR	0.07	NR	NR	NR	27.77	0.20	0.32	0.23

Legend: Column 1: Sample name (S: Survey sample, QS: Quantitative Survey sample, data for the sample both before and after poly(A) capture is shown), Column 2: Genotype identified in the sample by STM and used here as a reference sequence, Column 3: Total number of input NoV RNA copies (log_10_) used as input to poly(A) capture and library construction, Column 4: Ct value, Column 5: Total number of reads obtained before QC and trimming, Column 6: Total number of reads after quality trimming and filtering, Column 7: Number of NoV reads mapped to reference sequence indicated in Column 2, Column 8: NoV reads expressed per million reads in total, Column 9: NoV reads expressed per million reads in total, Column 10: B.uniformis reads expressed per million reads in total, Column 11: R. bromii uniformis reads expressed per million reads in total, Column 12: 16 sRNA reads expressed per million reads in total. The two rows at the bottom of the figure shows the calculated average fold change and standard deviation for all relevant measurements before and after poly(A) capture. ND: No data. NR: Not relevant.

### Outbreak analysis

**Figure 2:**
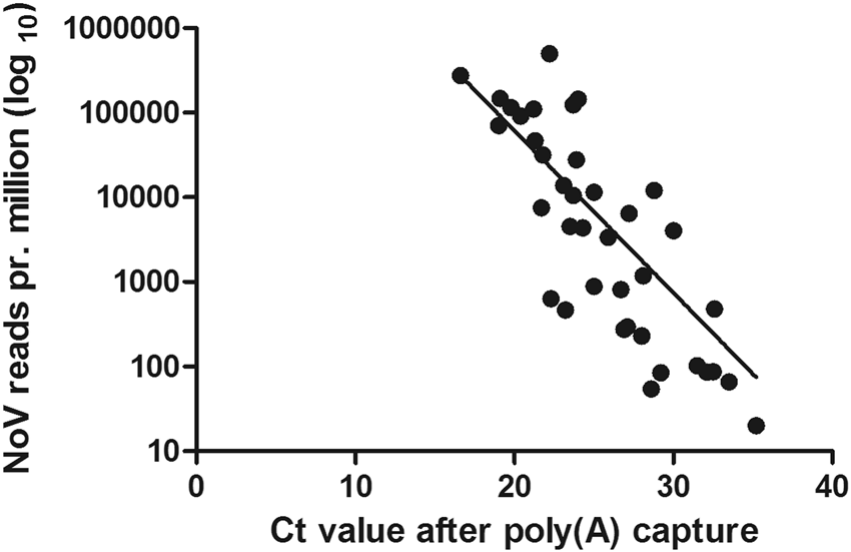
Relationship between Ct value (x axis) and the number of NoV reads per million reads (y axis, Log_10_). A robust regression analysis was performed in Prism (Robust Sum of Squares: 36.72).

**Figure 3:**
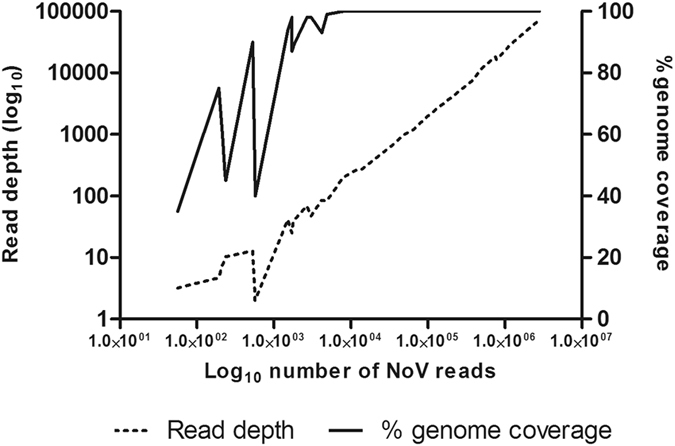
Relationship between the log_10_ number of NoV reads (X axis) and the percentage of full-genome coverage (Y axis: solid line) and the average read depth (Y axis: dotted line).

Samples from all eight outbreaks were subjected to the poly(A)-capture method and SMARTer library construction. A general linear trend was observed between the Ct values measured after poly(A)-capture and the number of reads obtained (Fig. 2), although a few samples deviated from this trend by containing a higher than expected number of NoV reads per million reads. Although full-genome coverage (>99%) was observed at ~4,800 NoV reads in total, equivalent to an average coverage of ~80 per sample (Fig. 3), sufficient sequence quality along the entire genome was only observed above ~11,000 reads with an average coverage of ~260.

### Assigning genotypes to outbreak samples

The first level of sequence comparison in an outbreak is the comparison of genotypes obtained from different persons in the outbreak. Complete NoV genotyping relies on sufficient sequence coverage in two regions: ORF1 (RdBp/pol) and ORF2 (Cap) for complete genotyping. Using the NGS approach, 14 complete and one partial genotype were detected in 15 samples from 6 of the 8 outbreaks (see Table 2) containing a single NoV genotype compared with 10 complete and five partial genotypes detected with the STM approach.

**Table 2:** Summary of sequencing, mapping and genotyping results of outbreak samples.

Sample name	Reads in total	Reads after QC/trim	Reference (genotype/acc. nr.)	Mapped reads	NGS-Pol/Capsid	Sanger Pol/Capsid	Average coverage	% of genome at any/quality depth
ob1-1	2,817,260	1,799,640	GI.P3_GI.3 (de novo)	778,531	+/+	−/+	15,617,24	100/100
ob1-2	2,663,142	1,855,781	GI.P3_GI.3 (de novo)	392,993	+/+	+/+	7,621,52	100/100
ob1-3	3,219,366	1,229,819	GI.P3_GI.3 (de novo)	102,072	+/+	+/+	1,985,00	100/99.97
ob1-4	3,548,472	1,259,481	GI.P3_GI.3 (de novo)	48,832	+/+	+/−	953,25	100/99.97
ob1-5	2,935,372	1,553,556	GI.P3_GI.3 (de novo)	13,242	+/+	+/−	260,26	100/99.66
ob2-1	6,127,782	2,586,676	GII.P21_GII.3 (KM198484)	1,807	+/+	+/+	35,78	89/69.21
ob2-2	2,524,586	1,138,380	GII.P21_GII.3 (KM198484)	213	+/+	+/+	4.51	60/24.62
ob3-1	2,696,616	1,316,156	GII.P21_GII.3 (EU921389)	235	+/+	+/+	4.58	45/23.27
ob3-2	2,685,158	1,516,777	GII.P21_GII.3 (EU921389)	231	−/+	+/+	4.27	47/22.47
ob4-1a	1,911,857	953,505	GII.P7_GII.6 (KM198534)	0	−/−	+/−	0	0/0
ob4-2a	3,100,594	1,596,062	GII.P7_GII.6 (KM198534)	3,811	+/+	−/−	74.06	89/68.57
ob4-1b	1,911,857	953,505	GII.Pe_GII.4_Sydney (JX459908)	48	+/+	+/−	0.95	41/3.36
ob4-2b	3,100,594	1,596,062	GII.Pe_GII.4_Sydney (JX459908)	0	−/−	−/−	0	0/0
ob4-1c	1,911,857	953,505	GII.Pg_GII.1 (HCU07611)	56	+/+	−/−	1.33	35/4.65
ob4-2c	3,100,594	1,596,062	GII.Pg_GII.1 (HCU07611)	0	−/−	−/−	0	0/0
ob4-1d	1,911,857	953,505	GI.Pb_GI.6 (JQ388274)	22	−/−	+/−	0.27	18/0
ob4-2d	3,100,594	1,596,062	GI.Pb_GI.6 (JQ388274)	35,464	+/+	−/−	682.34	100/97.37
ob5-1	2,911,740	2,830,406	GII.P4_New_Orleans_GII.4_Sydney (KJ685411)	191	+/+	+/+	3.52	75/21.8
ob5-2	2,652,197	2,554,030	GII.P4_New_Orleans_GII.4_Sydney (KJ685411)	11,523	+/+	+/+	263.01	100/99.6
Ob6-1a	4,777,166	4,448,206	GII.P7_GII.6 (de novo)	3,043	+/+	+/−	48.79	99/97.59
Ob6-2a	5,480,180	4,999,737	GII.P7_GII.6 (de novo)	4,849	+/+	+/−	85.45	99/93.58
Ob6-3a	5,530,180	5,148,480	GII.P7_GII.6 (de novo)	568	−/+	−/+	2.74	47/3.15
Ob6-4a	6,190,156	5,800,357	GII.P7_GII.6 (de novo)	1,705	+/+	−/+	26.75	99/93.55
Ob6-5a	5,126,044	4,097,985	GII.P7_GII.6 (de novo)	4,152	+/+	+/−	81.7	93/84.5
Ob6-6a	3,665,052	3,470,936	GII.P7_GII.6 (de novo)	1,713	+/+	−/−	27.18	89/74.16
Ob6-7a	8,212,260	5,991,822	GII.P7_GII.6 (de novo)	62,140	+/+	−/−	1,163.98	100/99.97
Ob6-1b	4,777,166	4,448,206	GI.P3_GI.3 (KJ196292.1)	116	−/+	−/−	0.28	12/0.52
Ob6-2b	5,480,180	4,999,737	GI.P3_GI.3 (KJ196292.1)	99	−/−	−/+	0.26	9/0.96
Ob6-3b	5,530,180	5,148,480	GI.P3_GI.3 (KJ196292.1)	968	+/+	−/+	15.68	88/61.56
Ob6-4b	6,190,156	5,800,357	GI.P3_GI.3 (KJ196292.1)	76	−/+	−/+	0.23	12/0.21
Ob6-5b	5,126,044	4,097,985	GI.P3_GI.3 (KJ196292.1)	37	−/+	−/−	0.13	7/0
Ob6-6b	3,665,052	3,470,936	GI.P3_GI.3 (KJ196292.1)	84	−/−	−/−	0.21	9/0.52
Ob6-7b	8,212,260	5,991,822	GI.P3_GI.3 (KJ196292.1)	292	−/+	−/−	1.06	14/1.92
Ob7-1	4,313,560	3,815,832	GI.Pb_GI.6 (JQ388274)	1,190,234	+/+	+/+	29,108.99	100/100
Ob7-2	5,597,036	5,525,067	GI.Pb_GI.6 (JQ388274)	2,797,949	+/+	+/+	72,731.53	100/100
Ob8-1	457,494	417,360	GII.P16_GII.4_Sydney (de novo)	1,514	+/+	−/+	38.76	93.88/86.21
Ob8-2	2,387,298	2,103,897	GII.P16_GII.4_Sydney (de novo)	2,673	+/+	−/+	67.22	97.81/90.46

Legend: Column 1: Sample name. Samples with identical numbers but different letters at the end are identical, but were mapped to different genotype reference sequence., Column 2: Total number of reads obtained before QC and trimming, Column 3: Total number of reads after quality trimming and filtering, Column 4: Genotype of NoV reference sequence used for mapping, Column 5: Number of reads mapped to reference sequence indicated in Column 4, Column 6: Pol or capsid genotyping results obtained with the NGS approach (genotype obtained: +, genotype not obtained: −), Column 7: Pol or capsid genotyping results obtained with the Sanger approach, Column 8: Percentage of reference sequence covered at either any fold coverage (to the left of the slash) or at >2 fold coverage (to the right of the slash). Consensus sequences generated from the >2 fold coverage were used for phylogenetic analysis. ND: No data.

Since it had been demonstrated by Real time PCR and STM that two NoV genogroups and several genotypes were involved in Ob-4 and Ob-6, HMM searches for additional genotypes was performed on *de novo* assembled contigs (See Materials and Methods). From sample Ob-4-1, 10,765 and from sample Ob-4-2 15,391 *de novo* assemblies were generated, of which 11 and 107 were identified as *norovirus* assemblies by the HMM search respectively. The candidate NoV contigs were further investigated by BLASTN and genotyping of the contigs, with subsequent reference based mapping which confirmed the presence of the following genotypes in the two samples: Ob-4-1: GII.Pg_GII.1 and GII.4_Sydney, Ob-4-2: GI.Pb_GI.6 and GII.7P_GII.6 (Table 2). Due to insufficient reads mapping to the GI.Pb_GI.6 reference in sample Ob-4-1, a valid phylogenetic comparison could not be performed, although a BLASTN of the consensus sequence generated from the 22 mapped reads indicated this to be GI.Pb_GI.6 as well. The HMM analysis also detected genotype GII.Pg_GII.1 in the Ob-4-1 sample which was not identified by STM.

From Outbreak 6, the following numbers of HMM hits were obtained out of the total number of *de novo* assembled contigs: Ob-6-1: 0 of 18,361, Ob-6-2: 2 of 1,928, Ob-6-3: 11 of 18,973, Ob-6-4: 6 of 34,959, Ob-6-5: 5 of 37,507, Ob-6-6: 4 of 14,250 and Ob-6-7: 1 of 43,308. Following the same procedure as described for Ob-4 lead to the identification of GII.P7_GII.6 and GI.P3_GI.3 in all seven samples. The GII.P7_GII.6 genotype was supported by a large number of reads in all samples except Ob-6-3 and could be compared phylogenetically, whereas the GI.P3_GI.3 genotype was only supported by a low number of reads in all samples. This also suggested that the two NoV genotypes were present in all the samples at variable concentrations.

Overall, support for the hypothesis of a common infection source by shared pol and cap genotypes for at least one genotype and in at least two different persons from the outbreak was obtained for seven of eight outbreaks with the NGS method and for five of eight outbreaks with the STM method. Similar genotypes were observed in several of the outbreaks, all of which were found to be different (identities: Ob-1/Ob-6: 84.4%, Ob-2/Ob-3: 99.8%, Ob-4/Ob-6: 84.2%, Ob-4/Ob-7/S-4: 97.6% to 98.8%, S-5/Ob-5: 97.3%).

### Phylogenetic analysis of outbreak samples

**Figure 4:**
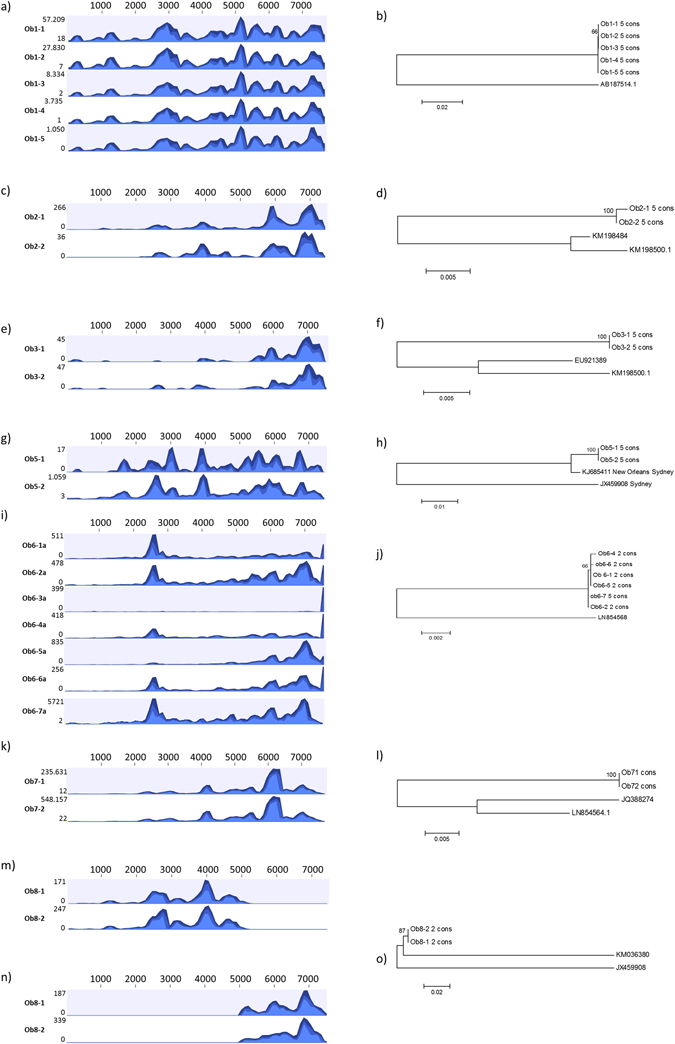
Genome coverage and phylogenetic comparison. Genome coverages are shown on the left side of the figure for the following outbreaks: Ob-1 (**a**), Ob-2 (**c**), Ob-3 (**e**), Ob-5 (**g**), Ob-6 (**i**), Ob-7 (**l**) and Ob-8 (**m** and **n**). The range of reads in the coverage plots are shown to the left of each coverage plot and the position on the used reference sequence is shown on the top. Phylogenetic trees of the consensus sequences are shown on the right side of the figure for the following outbreaks: from Ob-1 (**b**), Ob-2 (**d**), Ob-3 (**f**), Ob-5 (**h**), Ob-6 (**j**), Ob-7 (**l**) and Ob-8 (**o**) and relevant reference sequences.

Phylogenetic analysis was performed for all outbreaks, except for Ob-4 due to the absence of shared well-covered genotype reference sequences. Consensus sequences with the following maximum lengths were generated in CLCbio: Ob-1: 7666 nt, Ob-2: 1828 nt, Ob-3: 1226 nt, Ob-4: not analyzed, Ob-5: 1593 nt, Ob-6:7344 nt, Ob-7: 7697 nt, Ob-8: 6228 nt and used in a phylogenetic comparison. The genome coverage is shown in Fig. 4 for the individual outbreaks. The comparison included either the complete hypervariable P2 region (P2 region is 456 to 483 nt depending on genotype) for Ob-1, Ob-6, Ob-7 and Ob-8, and partial P2 region comparisons for Ob-2 (471 nt), Ob-3 (243 nt) and Ob-5 (291 nt). The phylogenetic analysis revealed that the NoV involved in Ob-3, Ob-5 and Ob-7 were 100% identical (Fig. 4f,h and l), whereas differences in the NoV genomes (Fig. 4b,d,j and o) were observed for: Ob-1 (1 nt difference; two samples had an A-residue at reference sequence position 3321 while three samples had a G-residue), Ob-2 (3 nt differences in the P2 region), Ob-6 (1 nt difference in ORF3 and several differences in the 3′ non-coding A rich part of the genome), Ob-8 (1 nt difference in the P2 region).

### Identifying a new recombinant

**Figure 5:**
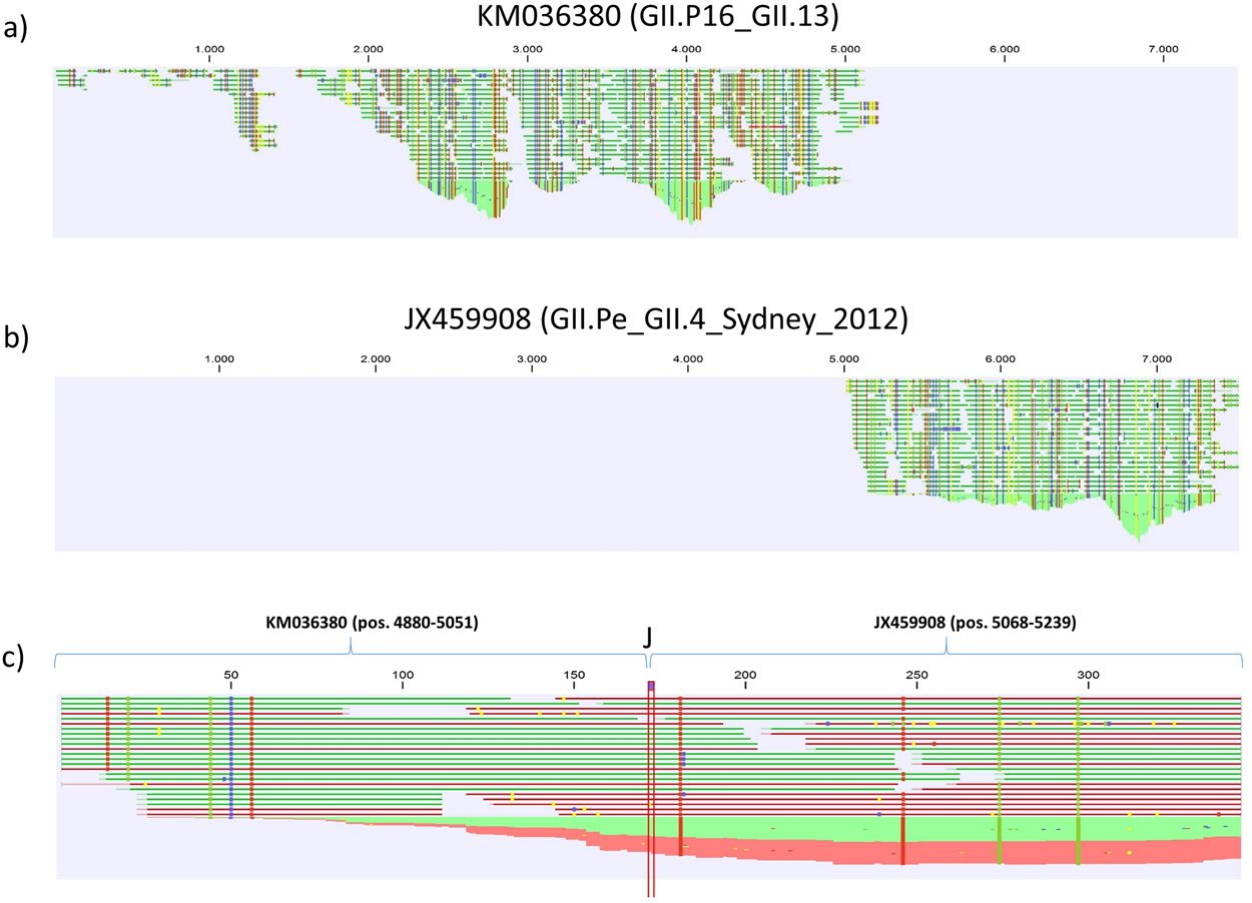
Read mappings for the recombinant NoV strain. (**a**) Mapping of reads to the reference sequence KM036380 (GII.P16_GII.13), (**b**) Mapping of reads to the reference sequence JX459908 (GII.Pe_GII.4_Sydney_2012), (**c**) Analysis of reads spanning the recombination junction region. This was performed by constructing an *in silico* reference sequence composed of pos. 4880 to 5051 of KM036380 joined with pos. 5068 to 5239 of JX459908 (the junction is marked with a “J”); reads were subsequently mapped to this reference sequence to identify junction-spanning reads.

When reads from the two samples from Ob-8 were mapped to the two reference sequences known to be present from the initial partial genotyping, a mutually exclusive distribution of reads was observed (Fig. 5a and b). In addition, reads that spanned the ORF1/ORF2 junction of an *in silico* generated reference sequence were observed (Fig. 5c), confirming that both these samples harbored a novel GII.P16_GII.4_Sydney recombinant.

## Discussion

The use of poly(A)-capture significantly enhanced the number of *norovirus* reads obtained from stool samples, allowing comparisons of full or near full (>85%) genome sequences from 4 outbreaks and partial genome comparisons in 3 outbreaks. In total, 14 complete and one partial genotype were detected in the 15 samples from the 6 outbreaks containing a single NoV genotype compared with 10 complete and five partial genotypes detected with the STM approach. In addition, additional genotypes (partial or complete) were identified with the NGS approach in the two mixed-genotype outbreaks samples (Ob4 and Ob6). STM generated more genotype information than NGS in four cases. In these cases, either none or a low number of NoV reads of mapped to the specific genotype, however none of the reads mapped to the ORF1 (pol) or ORF2 (cap) genotyping-regions. This showed that although the NGS method overall improved the genotyping results, some samples might be challenging due to low amounts of available virus RNA in combination with the random distribution of reads obtained. One way to reduce this problem would be to allocate a larger sequencing depth for especially samples with low amounts of virus.

The NGS derived consensus sequences used for phylogenetic comparison ranged from 1226 to 7692 nt (average 4800 nt) and included either the complete or a substantial proportion of the hypervariable P2 region. In comparison, STM only covers ~9% of the genome and does not include the P2 region. Therefore, even in the three outbreaks, in which only partial genomes were recovered, the data were found to significantly improve the molecular resolution of outbreaks.

Interestingly, minor nucleotide variations between sequences from different samples from three of the outbreaks were observed. Two of these differences were mapped to the P2 region, known to be highly variable^12, 34–36^ and a single nucleotide difference was observed between two groups of samples from a single epidemiologically linked outbreak (Ob-2). This challenge the 100% identity-paradigm used in general NoV outbreak investigations^12, 36^ that normally distinguishes only between identical and non-identical strains. Other studies have also questioned if these strict criteria should be maintained^37^, when comparing larger parts of the NoV genome.

NoV bioaccumulation in or adhesion to food items such as oysters and lettuce generates complex outbreak profiles including several genotypes^38, 39^, which require separate RT-PCR amplification steps if STM are used^39^. In this study, six NoV genotypes were identified in samples from two mixed outbreaks, three of which was supported by high genomic coverages (66% to 99% of the entire NoV genome). HMM improved the detection of genotypes by identifying a genotype (GII.Pg_GII.1) missed using STM. Although phylogenetic comparisons could not be performed for all genotypes due to varied sequence coverage of some genotypes in the samples, greater sequencing depth may circumvent this problem in future analysis. Interestingly, a mutually exclusive presence of genotypes was observed for three of the four genotypes identified in the two samples from Ob-4 and different relative abundances of the two genotypes found in Ob-6 was found for sample Ob-6-3 compared with the other samples. This could indicate differences in host exposure and/or susceptibility to different NoV genotypes in complex outbreaks.

A near-complete genome sequence of (>90%) a new GII.P16_GII.4_Sydney recombinant NoV was directly confirmed from the NGS data by using reads spanning the ORF1/ORF2 junction of the two different genotypes, showing that NGS can be used to distinguish between co-infection with different genotypes and new emergent recombinants.

This study was performed retrospectively on samples stored at −20 °C and previously analyzed by STM where samples had all been freeze-thawed at least twice, which may have resulted in some degree of degradation of the NoV. Five samples were excluded after poly(A)-capture, as a large increase (>5) in Ct values were observed, indicating fragmentation of NoV RNA. Therefore, for future applications of the present method, it will be of great importance to retain NoV RNA integrity until library preparation.

We have introduced a novel NoV enrichment NGS-based approach to investigate foodborne outbreaks without discriminating between genotypes. This method can be used directly to enrich other clinically important viruses in stool such as *enterovirus*es, or other positive-sense RNA viruses with a polyadenylated 3′ tail. Although the poly (A)-capture lead to a 3′ bias in sequencing depth, it allowed for a significant enrichment of NoV reads obtained from the samples. Future studies are required to test the efficiency of enrichment from other specimen types. Although the likelihood of obtaining complete NoV genomes is strongly dependent on NoV concentration in the sample, deeper sequencing would likely allow for retrieval of more NoV reads even in more scarce NoV samples. With common access to benchtop sequencers, we anticipate that NGS will soon become a definitive, non-discriminatory tool for viral infection control and serve to monitor both the evolution and spread of genotypes and enhance viral outbreak investigations.

## Materials and Methods

### Ethics statement

According to the “Danish Act on Research Ethics Review of Health Research Projects” this study does not require approval by the ethics committees, as it is considered a quality development/control project and does not analyze human sequences. This was confirmed by the Committees on Health Research Ethics for the Capital Region of Denmark in a specific waiver of approval (H-16019654).

### Sample material

Twenty-four NoV positive samples from eight different foodborne outbreaks (termed Ob1 to Ob8) were analyzed (Table 3). Five survey samples (termed S1 to S5) and 9 quantitative survey samples (termed QS1 to QS9) were analyzed with or without poly(A)-capture to assess the efficiency of this method. Five samples where Ct values increased >5 after poly(A)-capture vs. before were excluded from NGS analysis as they were considered to be too degraded.

**Table 3:** Line-list of eight norovirus-associated outbreaks in Denmark 2013–2015.

Outbreak (Ob)	Month and year	Suspected mode of transmission	Samples analyzed with NGS
Ob-1	March 2013	Person to person	Five
Ob-2	December 2013	Person to person	Two
Ob-3	February 2014	Person to person	Two
Ob-4	March 2014	Oysters	Two
Ob-5	June 2014	Person to person	Two
Ob-6	January 2015	Unknown (oysters or person to person)	Seven
Ob-7	April 2015	Unknown (fruit suspected)	Two
Ob-8	July 2015	Person to person	Two

Legend: Column 1: Outbreak number in chronological order, Column 2: Month and year of the outbreak, Column 3: Suspected mode of transmission, Column 4: Number of samples analyzed with NGS.

### Extraction of nucleic acids, poly(A) capture, real-time RT-PCR and *norovirus* typing

Nucleic acids were extracted from 10% stool suspensions (kept at −20 °C) using the MagNA Pure LC (Roche Diagnostics); poly(A)-capture was performed using a Dynabeads mRNA Purification Kit (Ambion Cat. No. 61006) according to the manufacturer’s instructions with modifications to use 100 µL input material and 26 µL Dynabeads. The concentration of nucleic acids was measured using 1 µL extract on a NanoDrop 1000 Spectrophotometer (NanoDrop Technologies). The presence of NoV Genogroup I and II was assessed using real-time multiplex PCR^40^ and genotyping was performed as described previously^40, 41^.

### Quantification of NoV RNA

A quantitative NoV GGI standard was obtained from ATCC (Quantitative Synthetic Norovirus G1 (I) RNA (ATCC® VR3234SD™; specification range (log_10_) 5–6 RNA copies/µL, of which the lower end range was used for the calculations. In addition, a previously published NoV GGII standard^42^ was obtained from collaborators at the Danish Technical University at a confirmed concentration of 5.19 (log_10_) ± 4.80 (log_10_) RNA copies/µL. Both standards were diluted in a fivefold 1:10 dilution series and analyzed in triplicates in the real time multiplex PCR (described above) alongside 9 NoV Quantiative Survey samples (QS1 to QS9; all both with and without poly(A)-capture). Analysis of real time data was performed in MxPro Mx3005 P v4.10, resulting in the following standard curves for GGI and GGII respectively: Y = −3.047xLOG(X) + 41.74; R^2^: 0.994 and Y = −3.090xLOG(X) + 41.18; R^2^: 0.971. Calculations of the amount of NoV genomes used as input in the extraction/capture and NGS analyses were also performed in MxPro.

### Preparation of samples for Illumina MiSeq sequencing

Single-indexed cDNA libraries were generated using the SMARTer Stranded RNA-Seq Kit (Clontech Inc.) in accordance with the manufacturer’s instructions. Fluorescent measurement of DNA concentrations in each library was performed using Qubit dsDNA BR and ssDNA assay kit (Thermo Fischer Scientific).

### Quality trimming and filtering

Sequences were imported into CLCbio’s Genomics Workbench (v. 8.5) with the removal of failed reads. Quality trimming within the workbench was performed using both a modified Mott trimming algorithm implemented (limit = 0.5) and by trimming reads containing more than two ambiguous nucleotides. Human sequence reads were removed by alignment to the *homo sapiens* hg19 reference genome (similarity fraction = 0.8).

### Reference based mapping

Quality-trimmed reads were mapped to reference sequences using the Mapping tool in CLCbio’s Genomics Workbench with default settings. NoV reads from all samples loaded on the same MiSeq run were mapped to all expected reference sequences. The following reference sequences were used for mapping of Miseq reads: JQ388274 (GI.Pb_GI.6), JX459908 (GII.Pe_GII.4_Sydney), KJ685411 (GII.P4_New_Orleans_GII.4_Sydney), DQ456824 (GII.P2_GII.2), EU921389 (GII.P21_GII.3), HCU07611 (GII.Pg_GII.1), JQ388274 (GI.Pb_GI.6), KJ196292.1 (GI.P3_GI.3), KM198484 (GII.P21_GII.3), KM198534 (GII.P7_GII.6) and the following reference sequences were used in the phylogenetic analysis: AB187514.1 (GI.P3_GI.3), KM198484 (GII.P21_GII.3), KM198500.1 (GII.P21_GII.3), EU921389 (GII.P21_GII.3), KJ685411 (GII.P4_New_Orleans_2009_GII.4_Sydney_2012), JX459908 (GII.Pe_GII.4_Sydney), LN854568 (GII.P7_GII.6), JQ388274 (GI.Pb_GI.6), LN854564.1 (GI.Pb_GI.6), KM036380 (GII.P16_GII.13), JX459908 (GII.Pe_GII.4_Sydney). In cases where no appropriate full-length reference sequence was available for mapping of reads, a *de novo* assembled sequence (see below) or a consensus sequence generated from the most similar full-length reference sequence available was used instead.

### *De novo* assembly

Reads from three outbreaks (Ob-1, Ob-6 and Ob-8) were mapped to *de novo* assembled reference sequences, as no well-matching and/or full-length reference sequences were identified in public databases. *De novo* assembled reads were generated using CLCbio’s assembler at default settings and with the fast mapping mode and a minimum contig length of 200 bases.

### Generation of consensus sequences

Consensus sequences were generated from mapped reads using the majority vote option and inserting N in places of ambiguity or missing data. Depth thresholds at >0, >2, or ≥5 reads were evaluated for sequence quality, and only high quality consensus sequences (average quality score ≥30, as calculated in CLCbio) were used for sequence comparison and phylogenetic analysis. The P2 region on the consensus sequence was defined as previously described^11^.

### Confirmation of genotypes

Genotypes from all mappings were confirmed by submission of consensus sequences to analysis at the Dutch National Institute for Public Health and the Environment (RIVM)’s NoV typing tool (http://www.rivm.nl/mpf/norovirus/typingtool) and/or BLASTN followed by genotyping of the best hits at RIVM.

### Hidden Markov model (HMM) building and searches

In total, 858 sequences matching the terms “norovirus” and “complete” at NCBI (accessed on April 25^th^, 2015) were downloaded and genotypes confirmed using the Dutch National Institute for Public Health and the Environment (RIVM)’s NoV typing tool (http://www.rivm.nl/mpf/norovirus/typingtool). From this set, 112 representative sequences were selected for hidden Markov model (HMM) building. Sequences were aligned in MAFFT v.7 (http://www.ebi.ac.uk/Tools/msa/mafft/) and a NoV HMM was built using HMMer 3.0^43^. HMM searches were performed among *de novo* assembled reads at default settings and identified assemblies evaluated by BLASTN and NoV typing at RIVM.

### Multiple alignment and phylogenetic analyses

Consensus and reference sequences were aligned in MAFFT and phylogenetic analyses were performed by maximum-likelihood with a generalized time-reversible (GTR) substitution model and a 1000 bootstrap replicates in MEGA 6.06^44^.
